# Optimization of ecological restoration efficiency in Qinghai-Tibet Plateau using the Cubist regression tree model: A study of environmental adaptability models

**DOI:** 10.1371/journal.pone.0335056

**Published:** 2025-11-12

**Authors:** Yuan Han, Wenhu Jin, Heng Liu, Wei Wang, Jie Ma, Wei Zhao

**Affiliations:** Xining Natural Resources Comprehensive Survey Center, China Geological Survey, Xining, China; Zhejiang Agriculture and Forestry University: Zhejiang A and F University, CHINA

## Abstract

This study addresses the inefficiencies in ecological restoration on the Qinghai-Tibet Plateau (QTP), particularly concerning prolonged vegetation restoration cycles, slow soil quality improvement, and difficulties in quantifying manual intervention measures. An integrated Cubist regression tree model is developed using ecological environment data from the QTP and multi-source environmental monitoring data from 2019 to 2023. The model combines a lightweight self-attention mechanism (SA) with bidirectional gated recurrent units (BiGRU) to enhance the accuracy and adaptability of restoration efficiency prediction. The SA mechanism dynamically adjusts environmental factor weights to strengthen nonlinear relationship capture capabilities, while the BiGRU learner optimizes temporal feature representation to accommodate spatiotemporal variability in restoration processes. Input factors include fractional vegetation cover (FVC), temperature, precipitation, soil moisture, and manual intervention measures (irrigation volume, planting density), with outputs being vegetation restoration rate and soil quality improvement effects. Experimental results demonstrate that the model achieves less than 5% error in vegetation restoration rate prediction, with correlation coefficients exceeding 0.90, and 96% accuracy in soil improvement prediction. Temperature and precipitation show contribution rates of 32% and 25%, respectively, while soil moisture and NDVI jointly contribute 25%. Prediction accuracy remains above 90% across different altitude zones, indicating strong regional adaptability. Notably, in areas with annual precipitation below 200 millimeters, every 10% increase in irrigation volume leads to approximately 15% improvement in vegetation survival rate. This study provides quantitative and operational intervention guidelines for plateau ecological restoration, enhances the evaluation efficiency of manual intervention measures, and has significant practical application value.

## Introduction

The ecosystem of the Qinghai-Tibet Plateau (QTP) is facing severe degradation, and this situation is seriously threatening its key functions as the “Asian Water Tower”—including water conservation and global climate regulation [1–3]. However, existing ecological restoration assessment models have shown obvious limitations in addressing this complex challenge. Therefore, this study urgently needs to develop more accurate and adaptable scientific tools to guide restoration work.

The QTP, affected by its unique alpine climate, fragile soil structure, and human activities, is experiencing an increasingly serious ecological degradation problem. This is specifically manifested in the decline of vegetation coverage, the intensification of soil erosion, and the sharp reduction of biodiversity [4]. Although China has continuously promoted ecological restoration projects such as converting farmland to grassland and artificial afforestation in recent years, the overall restoration efficiency is still unsatisfactory. Especially in high-altitude, arid, or freeze-thaw transition zones, the long vegetation restoration cycle and slow improvement of soil quality have become major bottlenecks restricting ecological reconstruction.

Part of the reason for this dilemma lies in the inadequacy of assessment and decision-making tools. Traditional ecological restoration assessment methods mainly rely on long-term field monitoring and empirical models. This approach makes it highly challenging to accurately quantify the actual effects of manual interventions or to capture the complex nonlinear coupling relationships among ecological factors [5]. Even the existing machine learning (ML) models [6–8], when applied to highly heterogeneous regions like the QTP in terms of geography, climate, and ecosystem, often expose problems such as poor spatial adaptability and insufficient prediction accuracy. They cannot effectively integrate multi-source heterogeneous data, nor fully consider the spatiotemporal dynamic changes of key factors in the ecological restoration process. Therefore, there is an urgent need for an intelligent modeling method that can integrate multi-source data and adjust dynamic factors. Meanwhile, this method can possess strong temporal feature representation capabilities to accurately assess restoration efficiency and optimize intervention measures.

To address these challenges, this study constructs a Cubist regression tree model that integrates Self-Attention (SA) and Bidirectional Gated Recurrent Unit (BiGRU). It aims to enhance the prediction accuracy and environmental adaptability of ecological restoration efficiency on the QTP. The objective is to achieve a quantitative evaluation of manual intervention measures and optimized decision support. The innovation of this study lies in proposing an integrated Cubist regression tree model incorporating lightweight SA and BiGRU (Cubist-BiGRU-SA), based on multi-source environmental monitoring data from the QTP during 2019–2023. The model introduces SA into the Cubist tree structure to dynamically learn and adjust the relative importance of environmental factors for ecological restoration. Meanwhile, it combines BiGRU learners to capture temporal evolution patterns during intervention processes. This method effectively improves the model’s generalization ability and prediction accuracy under the complex terrain and variable climate conditions of the QTP. Based on this, the study seeks to provide efficient and intelligent support tools for restoration projects in the QTP and other fragile ecological regions. Thus, it can promote the transformation of ecological restoration from experience-driven to data-driven and mechanism-driven approaches.

## Recent related work

### A review of the current research status of ecological restoration on the QTP

In recent years, with the proposal and implementation of ecological restoration concepts, the QTP’s restoration has attracted significant attention from researchers. Liu et al. (2022) [9] identified key priority areas for ecological restoration under different scenarios on the QTP, providing a scientific basis for restoration efforts. Their research emphasized that selecting priority protection zones proved crucial for enhancing restoration effectiveness across multiple intervention scenarios. Yu et al. (2023) [10] analyzed the carbon footprint impacts of grassland ecosystem restoration projects on the QTP. Results demonstrated that ecological restoration contributed to carbon emission reduction while improving carbon storage capacity in grassland ecosystems. Ma et al. (2023) [11] investigated multiple ecological effects and driving factors of restoration projects on the QTP. Findings revealed that ecological restoration not only improved FVC but also promoted biodiversity recovery and hydrological function restoration. Huang et al. (2024) [12] demonstrated that carbon loss caused by permafrost thawing on the QTP could be mitigated through ecological restoration. By increasing FVC, restoration projects effectively reduced temperature-induced carbon release. Li et al. (2024) [13] examined soil fungal community changes in restored grasslands. The study discovered significantly enhanced soil microbial diversity during restoration processes, with notable variations observed across different vegetation restoration approaches. Liu et al. (2025) [14] proposed multi-scale ecological restoration strategies to enhance water conservation capacity in the Zoige Wetlands of the QTP. Systematic restoration implementation substantially improved soil-water conservation and water resource management outcomes.

### Review of the application of ML in ecological modeling

Advances in data science and computing power have led to increasingly widespread applications of ML in ecological modeling. By automatically extracting patterns from complex data, ML has become a powerful tool in ecological research. Simon et al. (2023) [15] applied random forest (RF) analysis to enhance interpretability in ecological modeling, shifting focus from pure prediction to deeper explanation. By integrating ecological models with ML methods, the study improved the understanding of complex ecosystems and revealed causal relationships among variables. Zhang et al. (2023) [16] predicted spatiotemporal changes in ecological carrying capacity using ML and PLUS models. Results demonstrated ML’s effectiveness in capturing dynamic variations of ecological carrying capacity, providing reliable predictive tools for ecological management and planning. Liu et al. (2023) [17] employed ML methods to predict algal community structures. The algorithmic models accurately identified relationships between environmental variables and algal distribution, offering a scientific basis for aquatic ecosystem protection and management. Vázquez et al. (2024) [18] analyzed the ecological impacts of adolescent e-cigarette use through ML approaches. Data mining revealed ecological factors underlying health crises, providing evidence for public health policymaking. Najafzadeh et al. (2024) [19] established empirical formulas between water quality parameters and flow patterns using ML models. This approach enabled real-time assessment of aquatic ecological status, offering new perspectives for water resource management and pollution control. Ostovich & Klaper (2024) [20] combined ML with multiple ammonium algal cell imaging techniques to analyze complex phenotypes of plant-like organisms. Data analysis through ML provided deeper insights into plant ecosystems, facilitating ecological monitoring and conservation. Nguyen et al. (2025) [21] developed the HydroEcoLSTM Python toolkit, incorporating long short-term memory (LSTM) neural networks for aquatic ecological modeling. The toolkit provided automated visualization interfaces for hydro-ecological modeling, improving prediction accuracy. Mehmood et al. (2025) [22] assessed the ecological impacts of the “Ten Billion Trees” afforestation project using ML and spatiotemporal analysis. Research showed ML methods effectively monitored and predicted long-term ecological benefits of afforestation initiatives.

### Research gap and innovation

Existing studies have extensively explored ecological restoration on the QTP and the application of ML. However, several key gaps remain, especially in spatiotemporal data processing and the quantification of manual intervention measures. Current models generally have three major limitations. First, they insufficiently capture spatiotemporal dynamics, making it difficult to reveal long-term restoration patterns driven by altitude, seasonal changes, and climate variations; second, they face difficulties in quantifying manual intervention measures—most of them ignore the in-depth analysis of measures such as irrigation volume and planting density, leading to low efficiency of restoration input and output; third, they have limited adaptability and generalization ability, making it hard to effectively transfer across ecological sub-regions with huge internal differences on the plateau. To fill these gaps, this study proposes an innovative hybrid model that deeply integrates Cubist regression tree, BiGRU, and SA. The model uses a Cubist regression tree to extract the nonlinear synergetic effects between environmental factors and manual intervention measures; it leverages the bidirectional learning capability of BiGRU to deeply explore the temporal dependencies in the restoration process. Most crucially, it innovatively introduces SA, enabling the model to dynamically adjust the importance weights of various factors according to different spatiotemporal contexts; for example, it can automatically increase the weight of “irrigation volume” in dry seasons. This markedly enhances the model’s environmental adaptability and prediction accuracy. Through this design, the proposed model can accurately predict the vegetation restoration rate and soil improvement effect. Meanwhile, this model quantifies the synergetic effect between natural conditions and manual intervention measures, providing scientific and quantitative decision support for formulating “localized” ecological restoration strategies in different regions of the QTP.

## The ecological restoration method of the QTP

### Data source and preprocessing

The data used in this study are mainly derived from multiple monitoring platforms and field surveys on the QTP, involving three aspects: meteorological, remote sensing (RS), and artificial restoration data [23]. First, meteorological data are obtained from multiple meteorological stations within the QTP, covering day-by-day meteorological variables such as temperature and precipitation, and spanning the period from 2019 to 2023. These data allow an in-depth analysis of the impact of climate change on the ecological restoration process. Second, RS data are acquired by satellite RS technology and contain ecological variables such as soil moisture and FVC. The data provide large-scale and long-term environmental change trends, which are especially suitable for monitoring the dynamic changes of ecosystems. Lastly, the manual restoration data are obtained from field surveys in different regions of the QTP, which contain information on irrigation volume, planting density, vegetation survival rate, and soil physicochemical properties. The data sources and pre-processing results are exhibited in Table 1.

**Table 1:** Data sources and preprocessing procedures.

Data types	Data sources	Pre-processing methods	Processed data
meteorological data	Meteorological stations in the QTP	Interpolation methods are used to fill in missing values, seasonal adjustments are made, and outliers are removed.	Daily average temperature, precipitation, and standardized processing
RS data	Satellite RS images	Image correction, geometric registration, resampling, and temporal interpolation	Soil moisture, FVC, and standardized processing
Artificial restoration data	Field investigations and on-site measurement data	Data cleaning, extreme value removal, and standardized processing	Irrigation volume, planting density, and vegetation survival rate

To ensure data quality and consistency, this study implements rigorous preprocessing for data from different sources. First, meteorological data undergoes interpolation to fill missing values caused by equipment failure or extreme weather, followed by smoothing to remove potential outliers. Temperature and precipitation data receive seasonal adjustment to achieve stationarity. Second, RS data preprocessing includes image correction, geometric registration, and resampling. Since RS images may be affected by different sensors and acquisition times, orthorectification and spatial alignment are performed to ensure consistency with ground measurements.

For data fusion, this study primarily employs time-series analysis methods to integrate RS, artificial restoration, and meteorological data. While RS data offers high spatial resolution, its longer acquisition intervals necessitate temporal interpolation for alignment with meteorological data. Specifically, monthly or quarterly RS data is linearly interpolated to match the daily resolution of meteorological data. Additionally, though manual intervention measures exhibit strong locality and heterogeneity, regional zoning of restoration areas enables their integration with RS and meteorological data. Thus, a unified spatiotemporal dataset can be created that better supports model training and prediction.

### Construction and analysis of the environmental adaptability of the Cubist-BiGRU-SA model

**Fig 1:**
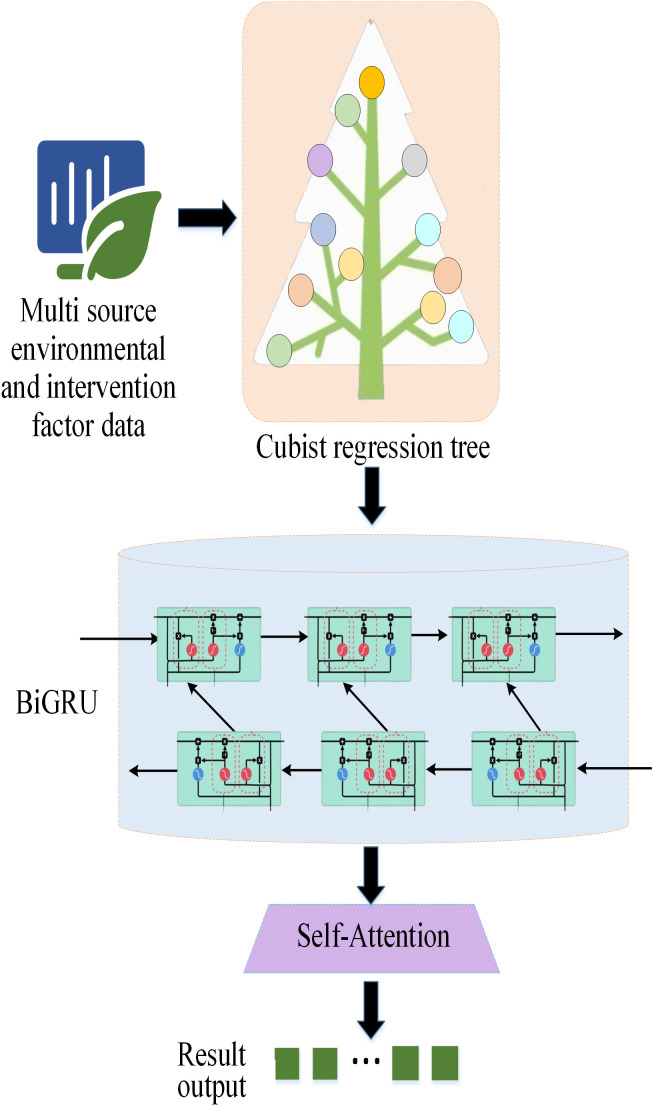
The architecture of the environmental adaptability of the Cubist-BiGRU-SA model.

This study proposes a novel hybrid prediction model, which deeply integrates Cubist regression tree, BiGRU, and SA, aiming to more accurately predict the complex ecological restoration dynamics of the QTP. The combination of these three components is not a simple stacking. Instead, it forms an analysis process with a clear division of labor and complementary advantages to overcome the limitations of single models in processing complex spatiotemporal data. Its core idea lies in using Cubist for efficient nonlinear feature extraction, capturing deep time-series dependencies through BiGRU. Finally, the SA is utilized to dynamically identify and focus on key time nodes, thereby significantly improving prediction accuracy and model interpretability. Fig 1 illustrates the Cubist-BiGRU-SA model’s architecture and data flow.

The specific integration logic of this hybrid model is as follows:

1) Feature Extraction Layer (Cubist): The input data is multi-dimensional time-series data including temperature, precipitation, FVC, irrigation volume, etc. At each time step, the Cubist regression tree first acts as a feature extractor. It divides the complex feature space into multiple sub-regions through a series of rules and fits a local linear model in each sub-region. This process effectively captures the nonlinear relationships and interaction effects between various input variables; it also converts the original features into a set of structured and more interpretable “rule-prediction” combined features.
2) Temporal Learning Layer (BiGRU): The structured features output by the Cubist layer at each time step are fed into the BiGRU network in chronological order. Through its forward and backward recurrent neural networks, BiGRU can simultaneously learn historical information (the impact of the past on the present) and future information (the implicit indication of future trends on the current state). Thus, BiGRU can comprehensively capture the long-term dependencies, seasonal fluctuations, and trend changes in the ecological restoration process.
3) Attention Aggregation Layer (SA): The output of BiGRU (i.e., the hidden state at each time step) is transmitted to the SA layer. By calculating the correlation weights between features at different time steps, this layer dynamically identifies the “critical moments” that contribute the most to the final prediction result. For example, when predicting vegetation restoration rate, the SA mechanism may automatically assign a higher weight to the time point when extreme drought occurs or large-scale irrigation is conducted. This enables the model to focus on decisive events and improve sensitivity to sudden changes and key processes.

Through the collaborative work of these three-layer structures, the model can gradually extract the core information that has the greatest influence on the ecological restoration results from the original, high-dimensional spatio-temporal data. As a result, it can achieve a complete and efficient analysis chain from feature engineering to time series modeling and then to the focus of key information.

**Fig 2:**
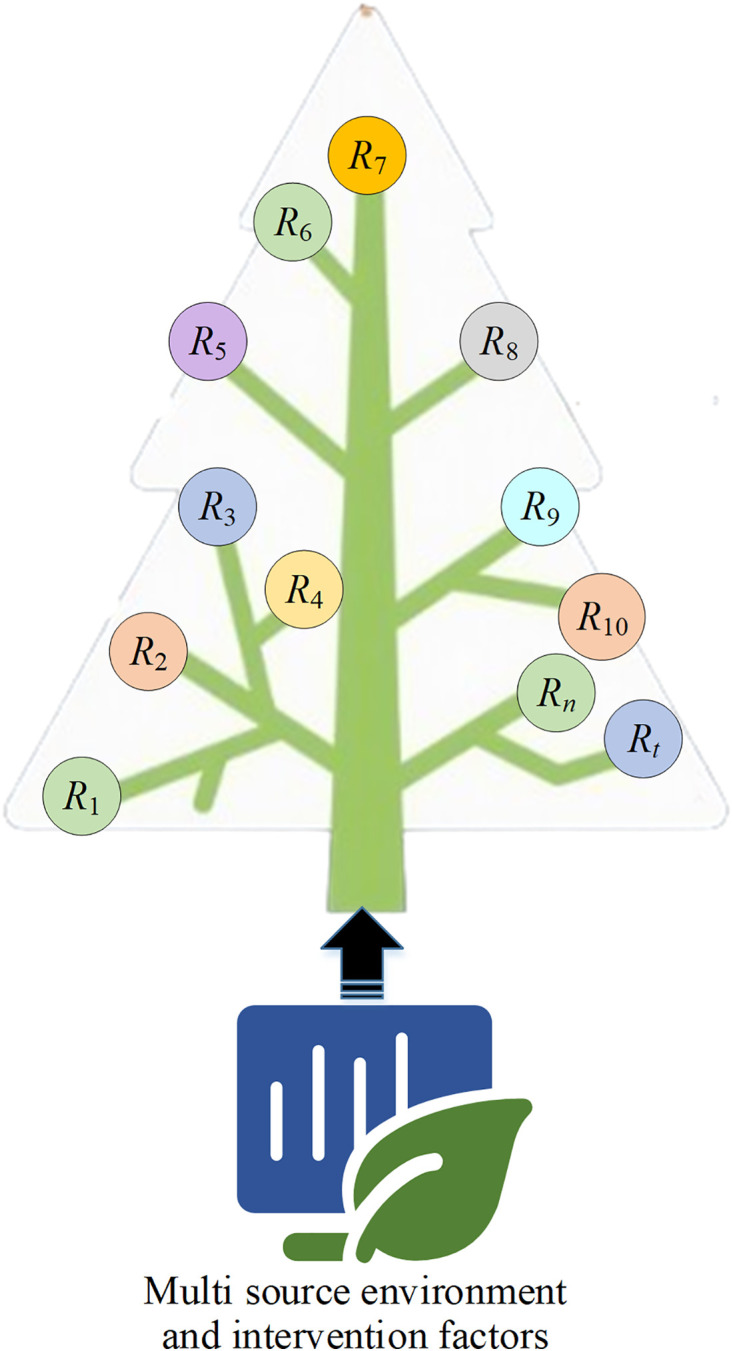
The data feature extraction module based on the Cubist regression tree.

In the data feature extraction layer, the Cubist regression tree effectively captures nonlinear structures and interaction effects among ecological data variables [24–26]. The process begins with rule-based partitioning of multi-source environmental and intervention factors (e.g., temperature, precipitation, soil moisture, irrigation volume). Then, it constructs local linear models within each partitioned region to obtain preliminary structural prediction relationships and combined features. The Cubist regression tree-based data feature extraction module is displayed in Fig 2.

Let the input sample be $x_{i}=\left[x_{i1},x_{i2},\cdots ,x_{id}\right]$ , Cubist first divides the feature space into multiple regions *R*_*j*_, and fits a linear model in each region, which can be represented as Equation (1):


$${\hat{y}}_{i}=\beta_{0}^{\left(j\right)}+\sum_{k=1}^{d}\beta_{k}^{\left(j\right)}x_{ik},\;x_{i}\in R_{j}$$ (1)

${\hat{y}}_{i}$ and *x*_*ik*_ are the predicted value and the *k*th input feature of the *i*th sample, respectively; $\beta_{k}^{\left(j\right)}$ denotes the linear regression coefficient in the *j*th regular region *R*_*j*_; *d* represents the dimension of the input variable.

Each sample, based on its characteristics, falls into a specific rule-based leaf node where the corresponding linear model performs prediction. This module outputs structured rule-based feature vectors $F=\left[f_{1},f_{2},\cdots ,f_{m}\right]$ as input for subsequent temporal modeling.

**Fig 3:**
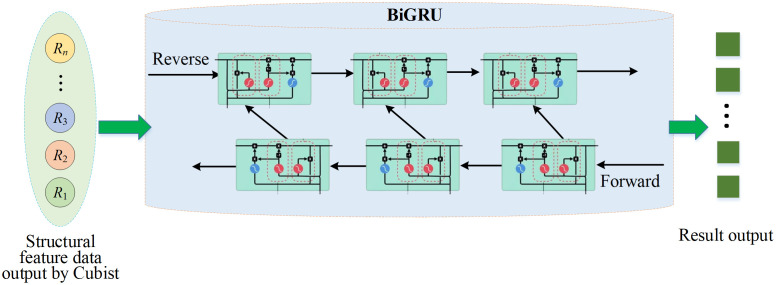
Schematic diagram of BiGRU applied to the temporal learning layer.

In the temporal learning layer, BiGRU serves as an improved recurrent neural network that learns long-term dependencies in sequential data along bidirectional temporal dimensions. It is particularly suitable for handling seasonal fluctuations and time-lag effects present in the QTP’s ecological data [27,28]. The application of BiGRU in the temporal learning layer is plotted in Fig 3.

The GRU of each time step *t* dynamically controls the information flow based on update and reset gates. Its basic calculation is shown in Equations (2)–(5):


$$z_{t}=\sigma (W_{z}x_{t}+U_{z}h_{t-1}+b_{z})$$ (2)

$$r_{t}=\sigma (W_{r}x_{t}+U_{r}h_{t-1}+b_{r})$$ (3)

$${\tilde{h}}_{t}=\tan h(W_{h}x_{t}+U_{h}(r_{t}⊙h_{t-1})+b_{h})$$ (4)

$$h_{t}=(1-z_{t})⊙h_{t-1}+z_{t}⊙{\tilde{h}}_{t}$$ (5)

*x*_*t*_ stands for the input vector at time *t* (i.e., the structural characteristics of Cubist output); *h*_*t*_ represents the current hidden state; ⊙ means the product of elements; σ is a sigmoid function; *W*_*z*_, *W*_*r*_, and *W*_*h*_ are weight parameters; *h*_*t*-1_ denotes the output of the former neuron; *b*_*z*_, *b*_*r*_, and *b*_*h*_ refer to offset vectors. BiGRU simultaneously calculates the hidden states of forward ${\vec{h}}_{t}$ and reverse ${\frac{\mathrm{\backslash buildrel}\mathrm{\backslash lower}3pt\text{\textbackslash{}(\textbackslash{}scriptscriptstyle\textbackslash{}leftarrow\textbackslash{})}}{h}}_{t}$, as expressed in Equations (6) and (7); then, they are spliced to obtain the final output, as given in Equation (8):


$${\vec{h}}_{t}=GRU_{fwd}\left(x_{t}\right)$$ (6)

$${\frac{\mathrm{\backslash buildrel}\mathrm{\backslash lower}3pt\text{\textbackslash{}(\textbackslash{}scriptscriptstyle\textbackslash{}leftarrow\textbackslash{})}}{h}}_{t}=GRU_{bwd}\left(x_{t}\right)$$ (7)

$$H_{t}=\left[{\vec{h}}_{t};{\frac{\mathrm{\backslash buildrel}\mathrm{\backslash lower}3pt\text{\textbackslash{}(\textbackslash{}scriptscriptstyle\textbackslash{}leftarrow\textbackslash{})}}{h}}_{t}\right]$$ (8)

Finally, the output sequence $\left\{H_{1},H_{2},\cdots ,H_{T}\right\}$ of bidirectional hidden state is obtained, which is the global temporal feature at each time step, providing context-aware representation for the attention layer.

In the attention aggregation layer, SA is employed to dynamically identify the time steps and variable dimensions with key influence in the temporal features, to improve the focus ability of the model on important patterns [29]. This layer mainly includes the generation and weight calculation of three groups of vectors: Query, Key, and Value, as follows:


$$Q=HW^{Q}$$ (9)

$$K=HW^{K}$$ (10)

$$V=HW^{V}$$ (11)

*Q*, *K*, and *V* refer to the matrix of “Query”, “Key”, and “Value” vectors; *H* means the timing representation matrix of BiGRU output; *W*^*Q*^, *W*^*K*^, and *W*^*V*^ represent the trainable parameter matrix. Then, the attention score matrices *A* and *Att*(*Q*, *K*, *V*) are calculated as Equations (12) and (13):


$$A=soft\max\left(\frac{Q\cdot K^{T}}{\sqrt{d_{k}}}\right)$$ (12)

$$Att(Q,K,V)=A\cdot V=soft\max\left(\frac{Q\cdot K^{T}}{\sqrt{d_{k}}}\right)\cdot V$$ (13)

The final output is the weighted fusion of the features of each time step, which can be written as Equation (14):


$$Att(Q,K,V)=\sum_{t=1}^{T}\alpha_{t}V_{t}$$ (14)

$\alpha_{t}$ refers to the importance weight of each time step, which determines the model’s attention to different ecological variables’ changes.

**Fig 4:**
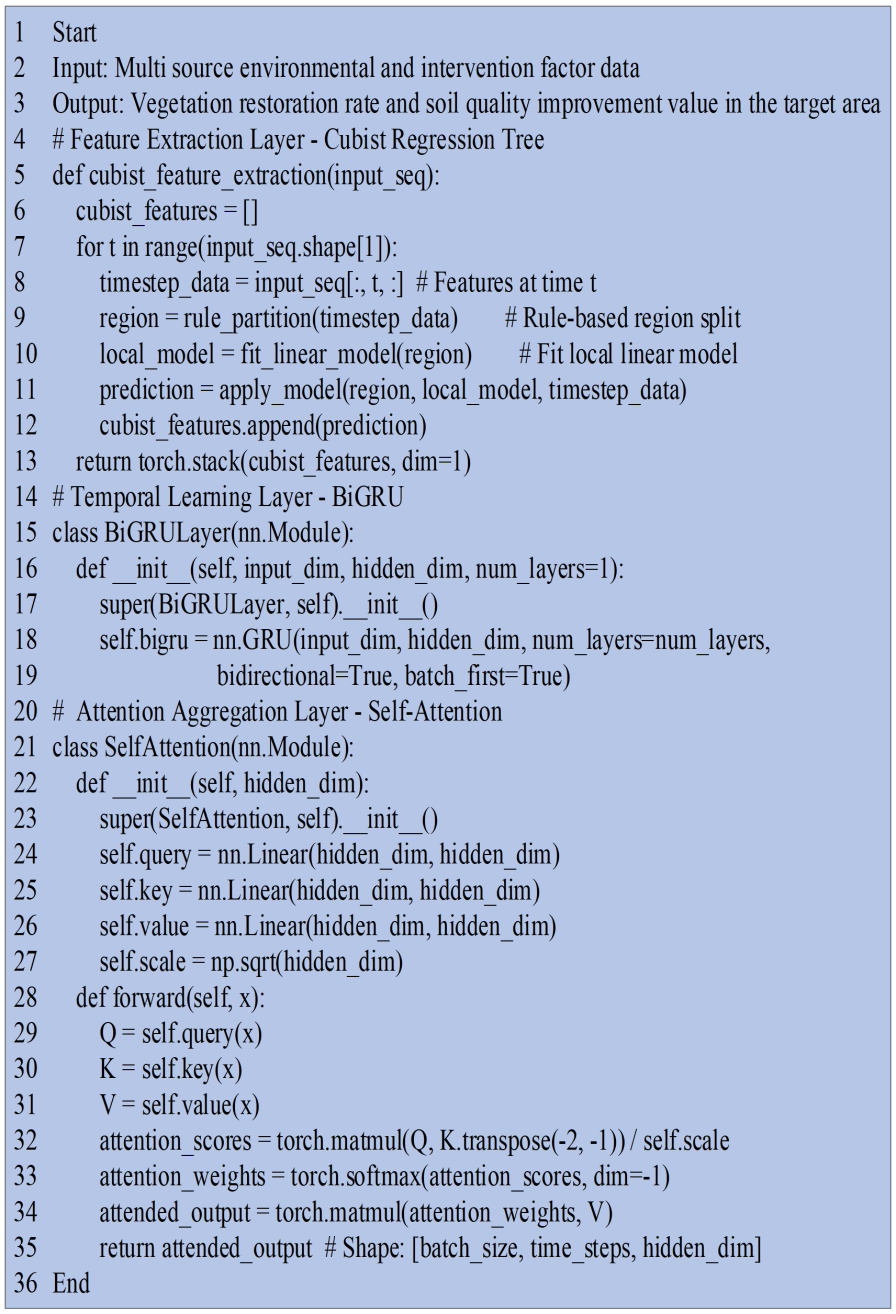
The process pseudocode for the environmental adaptability model based on Cubist-BiGRU-SA.

Through the collaborative operation of these three layers, the proposed model extracts complex nonlinear patterns (Cubist) and captures long-term temporal dependencies (BiGRU) while focusing on key variables and periods (SA). Hence, this model achieves high-precision modeling and quantitative prediction of ecological restoration efficiency in the QTP. The process pseudocode for the Cubist-BiGRU-SA-based environmental adaptability model is revealed in Fig 4.

### Experimental evaluation

This study designs a systematic experimental procedure to verify the effectiveness of the proposed Cubist-BiGRU-SA model in predicting ecological restoration on the QTP. The following sections detail the experimental setup, including data sources, preprocessing, model training protocols, and hyperparameter selection.

1) Data sources and preprocessing

This study adopts a multi-source spatiotemporal dataset covering the period from 2019 to 2023. The data mainly come from three sources: meteorological data, remote sensing observation data, and field survey data. The research area focuses on multiple typical ecological restoration demonstration zones on the QTP’s eastern edge. These sites cover different altitudes and ecological conditions to ensure the representativeness of the samples. All data are continuously collected over time at the same set of fixed monitoring plots, forming a high-quality panel dataset that provides a solid foundation for the model to learn temporal dynamic changes.

This study conducts strict preprocessing on the multi-source data to ensure their consistency and quality. The specific data sources, spatiotemporal frequency, preprocessing methods, and finally generated variables are summarized in Table 2. After integration and cleaning, a dataset containing 3,217 independent time-series samples is finally constructed for model training and testing.

**Table 2:** Data sources and preprocessing procedures.

Data type	Data sources	Original space-time frequency	Key preprocessing methods	Processed data
Meteorological data	National Level ground meteorological station network	Daily	Spatial interpolation to fill missing values, outlier removal, and normalization	Daily average temperature and cumulative precipitation
Remote sensing data	MODIS satellite products	16 days/8 days	Geometric correction, radiometric calibration, linear interpolation to match time frequency, standardization	FVC, soil moisture
Data on manual intervention measures	Project demonstration area field survey and annual records	Annual/Quarterly	Data cleaning, outlier removal, regional averaging, standardization	Irrigation volume, planting density

2) Model training and hyperparameter setting

This study divides the dataset into a training set (70%), a validation set (20%), and a test set (10%) in chronological order. Given the strong time-series nature of the data in this study, to prevent future information from leaking into the training process (which would lead to overestimated model performance), this study adopts a strict time-series split cross-validation strategy. This method ensures that the data used for validation or testing is always temporally later than the training data, thus enabling a more realistic evaluation of the model’s generalized prediction ability.

The model’s hyperparameters are not determined by automated methods such as grid search, but by a manual tuning strategy that combines domain experience and iterative experiments on the validation set. This method ensures model performance while taking computational efficiency into account. For example, this study finds that a two-layer BiGRU network is sufficient to capture complex temporal dependencies without causing severe overfitting. The finally selected key hyperparameters and their functional descriptions are detailed in Table 3.

**Table 3:** Key hyperparameter settings of the model.

Name of parameter	Setting value	Description and selection basis
Input time steps (T)	30	Length of the input historical time window (month)
Input variable dimension (D)	6	Including 6 items such as temperature, precipitation, and normalized difference vegetation index
Feature dimension of Cubist	16	Feature dimension of the structure combination of the Cubist output
BiGRU hidden layer dimensions	64	Balancing between model complexity and overfitting risk
BiGRU layers	2	Experiments show that a two-layer structure can effectively learn long-term dependencies
Dropout rate	0.3	Used to prevent overfitting and improve the model’s generalization ability
Learning rate	0.001	The manual optimization is achieved by observing the convergence of the loss function on the validation set.
Optimizer	Adam	An efficient optimization algorithm suitable for most scenarios
Batch size	32	The trade-off between GPU memory and training stability
Training epoch	100	Maximum number of iterations of the model

Additionally, an Early Stopping strategy is employed during training. The process automatically terminates when the validation loss shows no significant decrease for 10 consecutive epochs, thus reducing overfitting risks. All experiments are conducted on an NVIDIA A100 GPU platform using the PyTorch 2.0 framework for model construction and training.

The Cubist-BiGRU-SA model is compared with Cubist-GRU [30], RF-LSTM [31], BiGRU [32], and the model proposed by Nguyen et al. (2025) to comprehensively evaluate its performance in QTP’s ecological restoration prediction tasks. Multiple commonly-used evaluation metrics are selected, including Root Mean Square Error (RMSE), Mean Absolute Percentage Error (MAPE), coefficient of determination (R²), and prediction accuracy.

## Results and discussion

### Analysis of model prediction performance with different algorithms

**Fig 5:**
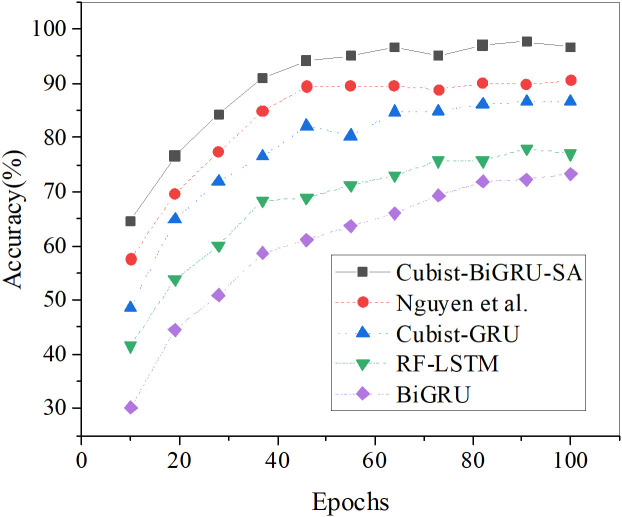
The prediction accuracy results of the vegetation restoration rate under different algorithms.

The prediction accuracy of the vegetation restoration rate of each algorithm with the increase in iterations is analyzed, and the results are demonstrated in Fig 5

Fig 5 reveals that the proposed Cubist-BiGRU-SA model consistently maintains superior prediction accuracy throughout all training epochs. This model achieves peak performance of 97.06% and 97.73% at the 82nd and 91st iterations, respectively, significantly outperforming other comparative models. While the model by Nguyen et al. (2025) reaches 90.57% at the 100th epoch, its overall accuracy remains consistently 6%−10% lower than the proposed model. The Cubist-GRU model shows stable performance during mid-to-late stages, peaking at 86.62% and outperforming both RF-LSTM and BiGRU. The RF-LSTM model exhibits slow overall improvement in prediction accuracy, with a maximum of 77.88%. The BiGRU model starts with low initial accuracy (30.12% at the 10th epoch) and eventually improves to 73.32%, yet still underperforms the proposed model by over 20 percentage points. Collectively, the Cubist-BiGRU-SA model demonstrates faster convergence speed and higher final accuracy, validating its advantages and robustness in multi-source environmental and anthropogenic variable integration modeling.

**Fig 6:**
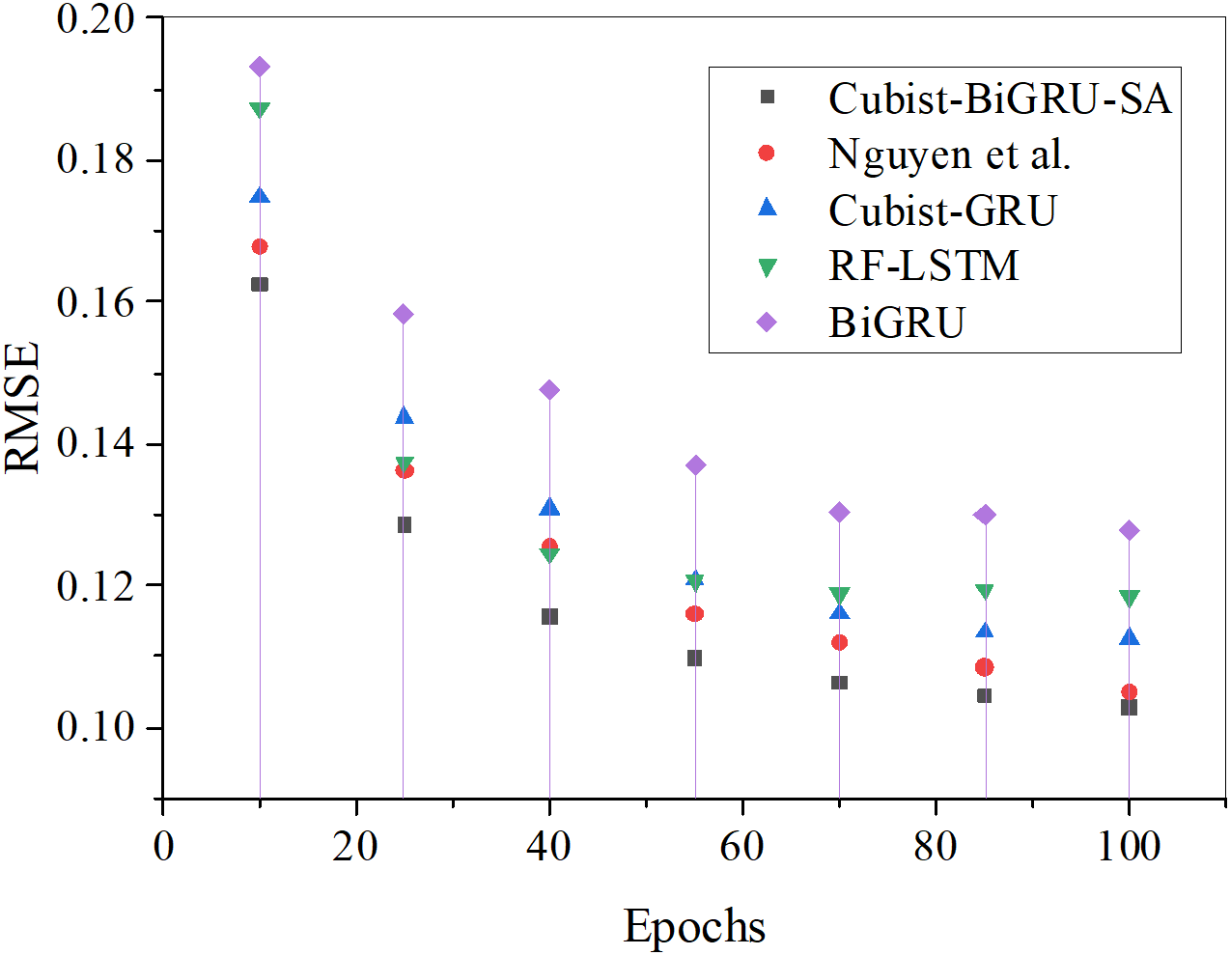
The predicted RMSE results for the vegetation restoration rate under various algorithms.

**Fig 7:**
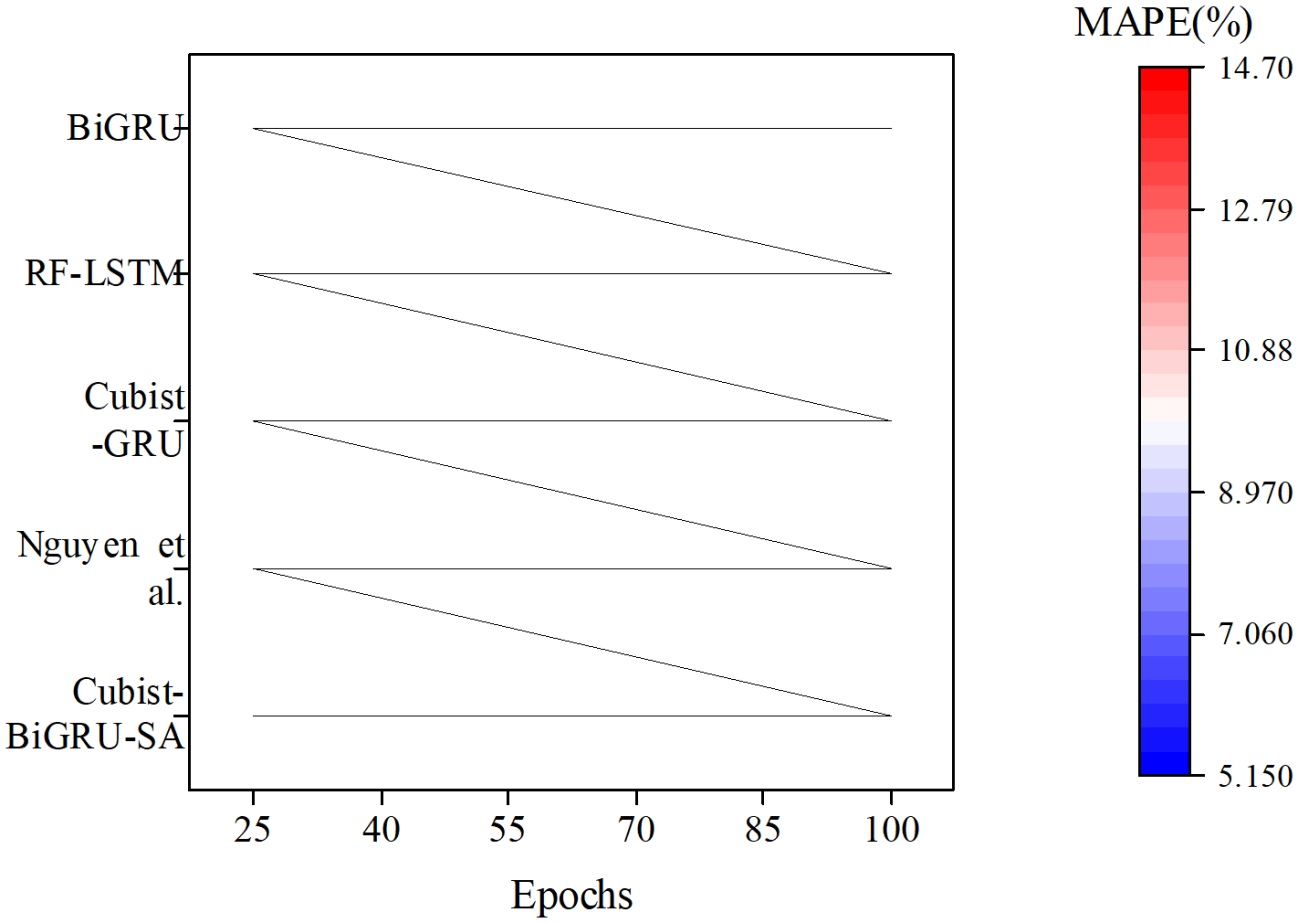
The forecasted MAPE results for the vegetation restoration rate with each algorithm.

**Fig 8:**
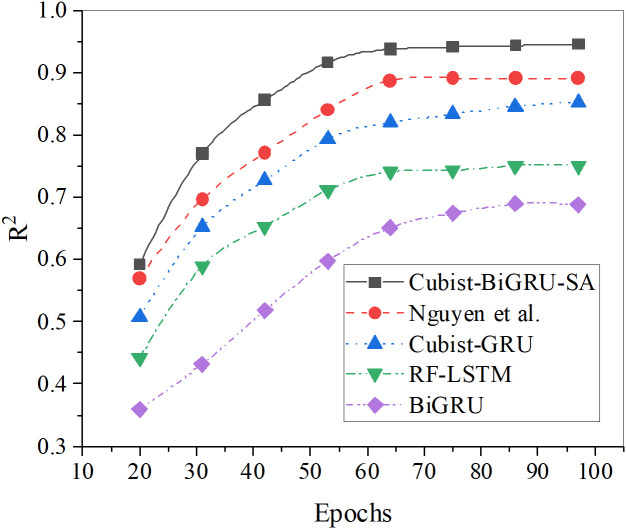
The predicted R² results for the vegetation restoration rate under diverse algorithms.

Further analysis of the predicted RMSE, MAPE, and R² results for the vegetation restoration rate across increasing iteration epochs for each algorithm is presented in Figs 6–8.

In Figs 6–8, comparative results of RMSE, MAPE, and R² demonstrate that the Cubist-BiGRU-SA model proposed in this study consistently exhibits optimal performance across all training epochs. Specifically, regarding RMSE, the model reaches 0.103 by the 100th epoch, remarkably outperforming Nguyen et al.‘s model (0.105) and other comparative models with the lowest error. This indicates minimal deviation between predicted and actual values. For MAPE metrics, Cubist-BiGRU-SA attains 5.22% at the 100th epoch, showing superior prediction accuracy and stability compared to Nguyen’s model (6.79%) and RF-LSTM (8.78%). Concerning R² evaluation, the proposed model achieves 0.9459 after 97 iterations, approaching perfect fit status (R² → 1), substantially exceeding BiGRU (0.689) and RF-LSTM (0.7513), demonstrating the strongest capability in explaining variable variance. Consequently, the proposed Cubist-BiGRU-SA model exhibits significant advantages over conventional deep learning models and existing research models in accuracy, stability, and interpretability; this validates its exceptional performance in modeling complex ecological restoration systems.

To comprehensively evaluate the performance of the proposed Cubist-BiGRU-SA model, this study compares it with multiple baseline models, including the standard Cubist model, the RF-LSTM hybrid model, and the standalone BiGRU model. This study uses RMSE, MAPE, R², and prediction accuracy as evaluation metrics. Prediction accuracy is defined here as the percentage of samples where the relative error between the predicted and true values falls within the range of ±10%. This metric is calculated on the test set, which encompasses the latest 2023 field survey data, thus ensuring the practical validity of the evaluation.

The final performance evaluation results of all models are detailed in Table 4. To test whether the proposed model’s superiority over other baseline models is statistically significant, this study conducts a paired t-test on the prediction errors of each model, and the p-values are also listed.

**Table 4:** Performance comparison and statistical significance test of diverse models on the test set.

Model	RMSE	MAPE (%)	R²	Prediction accuracy (%)	p-value (compared to Cubist-BiGRU-SA)
Cubist-BiGRU-SA (this study)	0.103	5.22	0.946	96.5	–
Cubist	0.145	8.15	0.851	82.3	< 0.01
RF-LSTM	0.178	8.78	0.751	77.9	< 0.01
BiGRU	0.195	10.05	0.689	73.3	< 0.001
Nguyen et al. (2025)	0.105	6.79	0.938	90.6	< 0.05

The results show that the proposed Cubist-BiGRU-SA model performs best across all evaluation metrics. It has the lowest RMSE and MAPE, indicating the smallest prediction error; its R² value is closest to 1, which means the model has the strongest ability to explain the data variance. Importantly, all p-values are less than 0.05, demonstrating that the performance improvement of the proposed model over all baseline models is statistically significant.

### Analysis of the roles of each factor in the model

**Fig 9:**
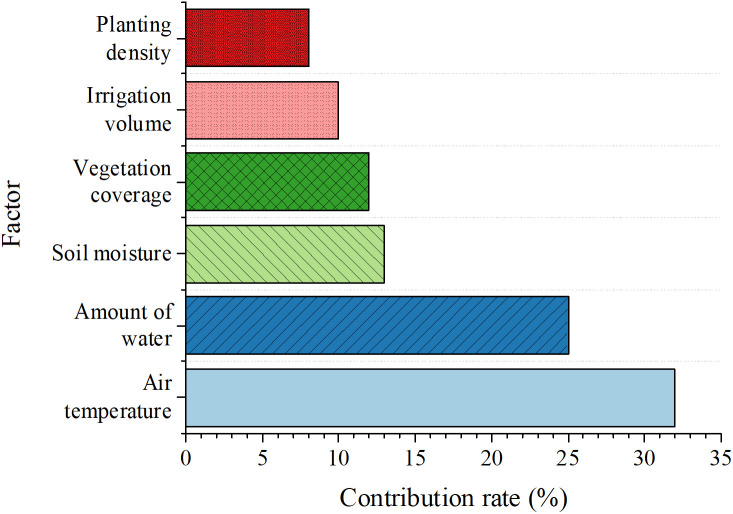
The global importance ranking of each input feature based on the mean absolute SHAP value.

To gain a deeper understanding of the model’s decision-making process and quantify the impact of each input feature on the prediction results, this study uses the SHapley Additive exPlanations (SHAP) method to analyze the trained model. SHAP values can reveal the magnitude and direction of each feature’s contribution to the prediction value of a single sample. Fig 9 provides a quantitative ranking of global feature importance by calculating the mean absolute SHAP value of each feature.

Fig 9 offers a clear quantitative ranking of global feature importance in the model’s prediction by calculating the mean absolute SHAP value of each input feature. This value measures the average impact magnitude of a single feature on the model’s output (vegetation restoration rate). The results demonstrate that natural climate conditions are the dominant factors determining the effectiveness of ecological restoration. Specifically, air temperature ranks first with the highest mean absolute SHAP value of 0.32, making it the most influential predictor; it is closely followed by precipitation with a value of 0.25. This indicates that heat and natural moisture conditions form the macro-environmental basis for plateau vegetation restoration. Next are key ecosystem state variables, including soil moisture (0.13) and FVC (0.12); their importance highlights the direct impact of the current ecological base on future restoration potential. Finally, although they rank lower than the main natural factors, the two manual intervention measures—irrigation volume (0.10) and planting density (0.08)—still show significant predictive power. This finding has important practical significance, as it quantitatively confirms that natural conditions set the upper limit of restoration. However, precise manual intervention measures (especially water supplementation and planting strategies) are effective levers to improve the efficiency of ecological restoration and achieve goals.

**Fig 10:**
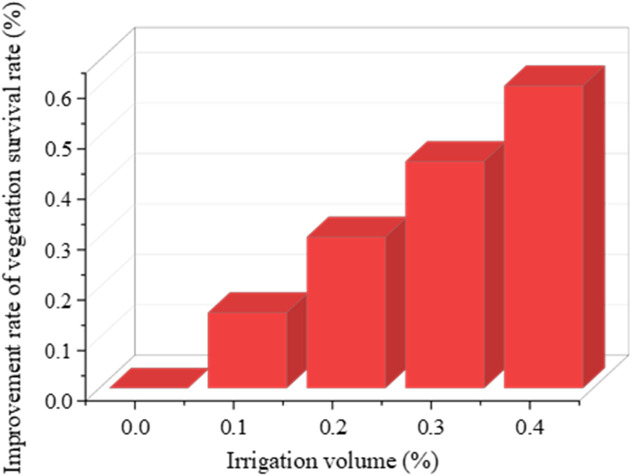
Relationship between irrigation volume and vegetation survival rate.

Further analysis of the relationship between irrigation volume and vegetation survival rate in arid zones is depicted in Fig 10.

Fig 10 illustrates that enhanced irrigation significantly boosts vegetation survival rates in areas receiving less than 200 millimeters of annual precipitation. Specifically, every 10% increase in irrigation volume corresponds to about a 15% enhancement in survival rate. When the irrigation volume reaches 40%, the survival rate can achieve 60%. These results quantitatively demonstrate that appropriate irrigation enhancement markedly improves ecological restoration outcomes in arid areas, providing measurable guidance for precision interventions.

To verify the generalization ability and stability of the model in the complex geographical environment of the QTP, this study divides the test samples (3217 × 0.1 ≈ 322 samples) into three regions according to altitude. The three include low-altitude (< 3500 meters), middle-altitude (3500–4500 meters), and high-altitude regions (> 4500 meters); meanwhile, the model’s prediction accuracy is evaluated for each region separately. The results are listed in Table 5.

**Table 5:** Prediction accuracy of the model in different altitude regions.

Altitude region	Sample size	Prediction accuracy (%)
Low-altitude region (<3500m)	97	97.2
Middle-altitude region (3500-4500m)	161	96.1
High-altitude region (>4500m)	64	95.8
Total	322	–

The results in Table 5 strongly prove the proposed model’s strong generalization ability and high stability in the complex and diverse geographical environment of the QTP. For this evaluation, this study divides the test set (containing 322 samples) into three representative regions by altitude: low-altitude (< 3500 meters), middle-altitude (3500–4500 meters), and high-altitude (> 4500 meters). The model exhibits excellent performance in all regions. Specifically, the prediction accuracy reaches 97.2% in the low-altitude region (with 97 samples), and 96.1% in the core middle-altitude region (the region with the largest sample size of 161); Even in the high-altitude region with the harshest ecological conditions and only 64 samples, its accuracy is as high as 95.8%. Most crucially, although the model’s accuracy shows an extremely slight decline as altitude increases and the environment becomes harsher, its performance remains at a high level of over 95%. This finding indicates that the model does not overfit to a specific environment; instead, it successfully learns the universal laws of ecological restoration, confirming its great potential for reliable deployment and application in different regions across the entire QTP.

### Discussion

This study successfully develops and verifies an innovative hybrid model integrating Cubist, BiGRU, and SA. This model demonstrates excellent accuracy (> 96%) and strong geographical adaptability in predicting the ecological restoration efficiency of the QTP. The study’s core contribution lies in achieving high-precision prediction and revealing the internal mechanism of the model’s decision-making through SHAP analysis. The results show that temperature and precipitation are the dominant natural factors determining the success of restoration. In contrast, manual intervention measures (such as irrigation volume) are key levers to improve restoration efficiency under specific conditions. This finding provides a solid quantitative basis for transforming ecological restoration management from an “experience-driven” model to a “data-driven” one, making it possible to formulate the most cost-effective intervention strategies with limited resources.

Compared with previous ecological modeling work conducted on the QTP, this study achieves significant methodological progress. The use of hybrid deep learning models to address complex environmental prediction tasks has become a cutting-edge trend, as demonstrated by Alharbi et al. in the field of intelligent agricultural water quality prediction [33]. This study aligns with this trend but addresses the unique challenges of ecological restoration through a specially designed architecture. Traditional statistical models struggle to capture nonlinear relationships, while single ML models have limitations. For example, although Cao et al. successfully applied ML to optimize water retention technologies for farmland soil [34], their research focused on the optimization of specific technologies. In contrast, the model proposed in this study aims to provide a more macro predictive framework to evaluate the comprehensive impact of multiple natural and human factors on the overall restoration effect (including soil quality). By leveraging Cubist to extract structured features, BiGRU to explore temporal dependencies, and SA to focus on the synergy of key events, the proposed model advances the technological frontier. Besides, it achieves a leap from single technology optimization to complex ecosystem outcome prediction.

Despite the encouraging results achieved by the proposed model, its limitations must be clearly recognized. First, the current model provides deterministic point predictions and fails to offer uncertainty estimation, which is crucial for risk assessment in practical decision-making. Second, the model’s performance highly depends on high-quality fixed-point monitoring data, and its scalability to larger regions faces challenges. This stands in sharp contrast to studies such as Odebiri et al. (2022), where the research team successfully used deep learning and publicly available Sentinel-3 satellite data to map soil organic carbon at the national scale; this highlights the limitation of this study in terms of data dependence. Additionally, the computational cost of the complex model may also be a factor to consider when conducting large-scale, high-frequency simulations.

These limitations also point out directions for future research. The top priority is to introduce uncertainty quantification methods, such as generating prediction intervals through Monte Carlo Dropout or quantile regression, to enhance the reliability of model outputs. Second, future work should explore integration with real-time, high-resolution remote sensing data streams to address the issues of scalability and data dependence. Drawing on the ideas of Odebiri et al., this integration can enable dynamic monitoring and prediction of broader regions [35]. Furthermore, the application of transfer learning technology—transferring knowledge learned from data-rich regions to new regions with sparse data—is a highly promising direction to reduce the model’s dependence on local data. Through these improvements, this study is expected to further develop the current model from a powerful analytical tool into a comprehensive, robust, and operational intelligent decision support system for ecological restoration.

## Conclusion

This study constructs a multi-level spatiotemporal modeling framework (Cubist-BiGRU-SA) that integrates Cubist regression tree, BiGRU, and SA. This model remarkably outperforms existing methods in the prediction accuracy of vegetation restoration rate and soil quality improvement (exceeding 96%). More importantly, the model provides key decision support for formulating scientific and practical ecological restoration plans. The proposed model can convert complex ecological data into clear and actionable guidelines by accurately quantifying the synergetic effects of natural factors and manual intervention measures (such as irrigation volume and planting density). For example, it can specifically answer practical questions like “In a specific arid area, what percentage of increase in vegetation survival rate can be achieved by increasing irrigation volume by 10%?” This enables ecological restoration work to shift from the traditional “experience-driven” model to a “data-driven” one, thus greatly improving the targeting and effectiveness of restoration measures as well as the efficiency of fund utilization. It provides a powerful scientific tool for achieving accurate and efficient restoration goals in the vast and diverse ecological environment of the QTP.

Despite the excellent performance of the model, it still has the following limitations. First, the model’s adaptability under future extreme climate change scenarios needs to be verified, and it fails to cover all key intervention factors, such as grazing activities and land policies. Meanwhile, the current method’s dependence on high-quality fixed-point data also poses challenges to its scalability to larger regions and brings corresponding computational costs. Therefore, the direction of future research is multi-dimensional. Future efforts should be made to explore integration with large-scale real-time remote sensing monitoring and efficiently apply the model to other ecologically fragile areas with sparse data through transfer learning. More importantly, methods such as causal inference should be introduced to deeply reveal the internal mechanism of intervention measures, thereby upgrading the model from an accurate prediction tool to a more in-depth decision support system.

## Supporting information

S1 File Data Packet. (ZIP)
