# Supplementary material for: Optimization of ecological restoration efficiency in Qinghai-Tibet Plateau using the Cubist regression tree model: A study of environmental adaptability models
Source: PLoS One. 2025 Nov 12;20(11):e0335056. doi: 10.1371/journal.pone.0335056 (PMC12611123; doi:10.1371/journal.pone.0335056)
Supplement: S1 File — (ZIP) [file pone.0335056.s001.zip › Model code and configuration description.docx]

Model code and configuration description

The model consists of three core layers: **a Cubist-based feature extraction layer, a BiGRU-based temporal learning layer, and a Self-Attention-based aggregation layer.** Finally, the vegetation restoration rate and soil quality improvement value are predicted through the output layer.

**1. Feature Extraction Layer - Cubist** (**cubist_feature_extraction** function): This layer simulates the Cubist regression tree process, performing rule-based partitioning and local linear modeling on the input multivariate time series data, and outputs structural features. It accepts raw ecological variable sequences with an input shape of [batch_size, time_steps, num_features] and outputs structural prediction features (combining both rule-based and linear fitting characteristics)

**2. Temporal Modeling Layer - BiGRU** (**BiGRULayer** class): This bidirectional gated recurrent network structure captures long-term dependencies and seasonal evolution patterns in time series data. Input: The structured Cubist features. Output: Hidden states at each time step (forward + reverse concatenation).

**3. Attention Aggregation Layer - Self-Attention** (**SelfAttention** class): This layer implements weighted modeling of time steps through dynamic weight allocation, emphasizing important temporal features. Using Query, Key, and Value vectors, it calculates attention scores and performs a weighted combination, thereby enhancing the model's sensitivity to critical variables and periods.

**4. The output layer (OutputLayer class):** The temporal features are averaged, pooled, and sent to the fully connected layer to output two continuous variables. Final output: [vegetation restoration rate, soil quality improvement value] and others.

Hyperparameter setting:

| **Parameter name** | **Meaning and description** |
| --- | --- |
| cubist_dim | The feature dimension after Cubist feature extraction is 16. |
| hidden_dim | The hidden layer dimension of BiGRU, 64 by default, can be adjusted according to task complexity. |
| num_layers | The number of GRU layers, which is 1 by default, is suitable for small sample ecological temporal modeling. |
| input_data shape | [batch_size, time_steps, num_features], such as [16, 30, 6]. |
| output | [batch_size, 2] represents the predicted vegetation restoration rate and soil quality improvement value. |
| optimizer | It is a commonly used Adam optimizer. The learning rate can be set to 1e-3 to 1e-4. |
| batch_size | It is 16-64, depending on the GPU video memory and the amount of data. |
| dropout | If the model is overfitted, dropout=0.3 can be added to BiGRU or attention. |
